# Cognitive Factors for Predicting Treatment Response in Schizophrenic Patients: One-Year Follow-Up Study

**DOI:** 10.4306/pi.2008.5.2.106

**Published:** 2008-06-30

**Authors:** Yong-Ku Kim, Ae-Ra Lee, Ji-Won Hur, Ho-Kyung Yoon, Bun-Hee Lee, Young-Hoon Ko

**Affiliations:** Department of Psychiatry, Korea University Ansan Hospital, Ansan, Korea.

**Keywords:** Cognition, Predictor, Schizophrenia, Treatment response, Prognosis, Wisconsin Card Sorting Test

## Abstract

**Objective:**

This study was conducted to investigate the cognitive factors that can longitudinally predict the response to treatment in patients with schizophrenia.

**Methods:**

The subjects were 49 patients with schizophrenia who were newly hospitalized in an acute psychiatry ward and had not been treated with medication for at least 8 weeks prior to the study. The symptoms and cognitive functions of the patients were evaluated at baseline before treatment (T0), at eight weeks after treatment (T1), and one year after treatment (T2). Clinical symptoms were assessed using the PANSS, and cognitive functions were estimated using the Vigilance Test, Cognitrone Test, Wisconsin Card Sorting Test (WCST), and the Korean version of the Memory Assessment Scales (K-MAS).

**Results:**

The patient group showed marked impairments in cognitive function when compared to the normal group, but the patients' clinical symptoms and cognitive functions improved after drug treatment. The patients also showed consistent improvement in verbal and nonverbal memory function as time progressed. Furthermore, there was a significant correlation between clinical symptoms and cognitive functions in the patient group. The cognitive variables that best predicted treatment response and prognosis were total errors on the WCST and immediate list recall component of the K-MAS. It was also shown that the number of total errors on the WCST was a better cognitive predictor than the number of errors in immediate recall.

**Conclusion:**

The results of the present study show that the neurocognitive functions of patients with schizophrenia can be stabilized with treatment intervention, that treatment response is related to improvement in cognitive function, and that cognitive domains, especially executive function, can predict treatment response and prognosis in patients with schizophrenia.

## Introduction

A number of studies have considered cognitive deficits to be an essential feature of schizophrenia.^1^ Several studies have reported that cognitive functioning deteriorates over time in patients with schizophrenia,^2^-^5^ while other studies have stated that cognitive functioning can change over time and significantly improve as clinical symptoms stabilize.^6^-^8^ Two hypotheses have been proposed regarding whether cognitive performance in schizophrenia is stable over time or fluctuates throughout the course of the illness. The trait hypothesis suggests that abnormalities in cognitive functioning are relatively stable and are caused by structural abnormalities in the brain, whereas the state hypothesis proposes that cognitive deficits are changeable because they are based on a neurochemical or neurophysiological disturbance. It is, however, more reasonable to combine these two hypotheses and suggest that cognitive function declines as schizophrenia progresses but that it can change according to the acute deterioration and treatment of psychosis.^9^

The relationship between symptoms and cognitive impairment is also an issue. A few studies have noted that although cognitive function in patients with schizophrenia did not improve significantly during treatment, the patients' clinical symptoms could be improved,^10^,^11^ while other studies reported that specific symptoms or groups of symptoms are related to a particular cognitive impairment.^12^,^13^ Censits et al.^14^ observed cognitive deficits in a group of schizophrenia patients over a period of 19 months and concluded that cognitive deficit patterns are stable, while improvement in clinical symptoms, especially negative symptoms, is significantly correlated with enhancement in neuropsychological performance. Hoff et al.^15^ also found a significant correlation between improvement of cognitive functions and decline of positive symptoms.

A number of studies have also investigated the course of schizophrenia and sought to identify predictors of treatment outcome. Some studies suggested that neurocognitive tests could be used to predict prognosis or the future effect of treatment in patients with schizophrenia.^16^-^19^ Robinson et al.^18^ concluded that the treatment response of schizophrenia patients could be predicted by using attention and motor vulnerability. Smith et al.^17^ demonstrated that poor performance on the Trail-Making Test predicted poor treatment response, and Chen et al.^19^ stated that perseverative errors on the Wisconsin Card Sorting Test (WCST) predicted the possibility of relapse. The identification of cognitive predictors would aid in the development of treatment strategies for improving the progression of the illness. Moreover, it is beneficial to examine cognitive factors, as these have been closely linked to the enhancement of psychosocial functions, such as social problem solving, social skill learning, and adjustment to the local community.^20^,^21^

The aims of this study were to explore longitudinal clinical symptoms and cognitive functioning in patients with schizophrenia, to identify specific cognitive domains in patients with schizophrenia and to clarify whether cognitive functioning deteriorated over time or whether it improved and stabilized after treatment.

## Methods

### Subjects

Forty-nine of the schizophrenia patients who were recently admitted to our acute psychiatric unit met the Diagnostic and Statistical Manual of Mental Disorders IV (DSM-IV) criteria for schizophrenia and were neuroleptic-free for at least 8 weeks (including 23 neurolepticnaive, first onset patients) were included in this study. The patients were assessed using the Structured Clinical Interview for DSM-IV Axis I Disorders (SCID-I).^22^ The exclusion criteria included: a) patients with an organic mental disorder, a neurological disorder, epilepsy, brain damage, mental retardation, or a history of substance abuse; b) patients who had corrected eyesight under 0.5 or who had impaired hearing or motor function; c) patients who had an impulse regulation problem or who were non-cooperative. To rule out the effects of education and age on neurocognitive assessment, the subjects were limited to high school graduates between the ages of 18 and 49 years. A total of 49 patients were included in the study, and all participants provided written informed consent.

Forty-nine patients were included in the reassessment analysis after an eight-week interregnal period, and 34 of these were re-examined after one year. At the 8-week point, 27 patients were medicated with risperidone (mean 6 mg; range, 4-10 mg), 16 patients with amisulpride (mean, 600 mg; range, 400-1,200 mg), and 6 patients with aripiprazole (mean, 20 mg; range, 15-30 mg). Thirty-four patients completed the one-year follow-up, and 20 of them were being treated with risperidone (mean 5.5 mg; range, 2-8 mg), 10 patients with amisulpride (mean, 450 mg; range, 200-800 mg), and 4 patients with aripiprazole (mean, 20 mg; range, 15-30 mg).

A total of 100 normal control subjects were recruited. The normal controls were matched with the patients for gender, age and education. They were recruited though advertisement and were screened using the SCID-I, non-patient version. None of the patients had a medical/psychiatric illness or family history of mental illness in first- and second-degree relatives. They received a modest fee (70 dollars) for completing the interview and neuropsychological tests. Table 1 shows the demographic data of both groups. This study was approved by the Institutional Review Board of Korea University, Ansan Hospital.

### Assessments

The neuropsychological tests were assessed by a clinical psychologist who had more than one year of experience with the tests. Cognitive evaluations were conducted three times before antipsychotic treatment (T0), after eight weeks (T1) and one year (T2) of receiving antipsychotic drugs, and included the Vigilance Test, Cognitrone Test, Korean version of Memory Assessment Scales (K-MAS), and WCST. At the same time, the patients' clinical symptoms were assessed using the Positive And Negative Syndrome Scale (PANSS).^23^

The Vigilance test is a subtest included in the Vienna Test System (PC/S Vienna Test System)^24^,^25^ that is used to measure sustained attention and vigilance. The Cognitrone test, another subtest of the Vienna Test System, is a complex task evaluating attention, perception, cognitive flexibility, figure perception, short-term memory, and stress endurance.

The K-MAS is the Korean version of the MAS, as translated and standardized by Lee et al.,^26^ and it is reported to have a high subtest reliability (.85-.91) and test-retest reliability (.62-.88). The MAS,^27^ originally referred to as the Vermont Memory Scale (VMS), is used to assess memory function in normal and clinical groups.^28^ It has been reported to compensate for the limitations of other memory tests.^29^ This test evaluates three domains of memory: 1) attention, concentration, short-term memory; 2) learning and immediate memory; and 3) delayed memory.

The WCST is a representative neuropsychological test used to measure executive function. The WCST requires the development and maintenance of precise problem solving strategies under various test conditions. This study uses the WCST Computer Version-2 developed by Heaton.^30^

### Statistical analysis

First, the neurocognitive function of the patients was compared with that of the normal group. Second, the effect of drug treatment on cognitive functioning and clinical symptoms of the patients was evaluated, and cognitive function was correlated with clinical symptoms. Third, the patient group was divided into responders and non-responders on the basis of clinical symptom relief in order to identify cognitive variables that may discriminate between responders and non-responders after one year. A 20 percent improvement in PANSS score was used as the criterion for improvement in clinical symptoms in accordance with the report by Stip et al.^31^ Statistical analysis was carried out using SPSS 10.0 and statistical methods, such as repeated-measure ANOVA, t-test, correlational analysis, and discriminant analysis.

## Results

### Cognitive functioning in patients with schizophrenia and normal controls

The cognitive function scores of the patients and normal controls were compared (Table 2). The two groups showed significant differences in all scores of cognitive function, except for the short-term memory component of the K-MAS and the total number of correct categorizations in the WCST. The results showed that cognitive function was remarkably degraded in the patient group.

### Changes in cognitive functions and clinical symptoms in schizophrenia

The changes in the clinical symptoms and cognitive functioning of the patients were examined during the period of T0-T2. The patients showed significant differences in all clinical symptoms, including positive, negative, and general symptoms, as well as PANSS total scores during T0-T2 (F=35.68, p<.001; F=7.76, p<.001; F=19.22, p<.001; F=24.91, p<.001).

Clinical symptoms at T0-T1 and T0-T2 were compared respectively. The results indicated that the total score, positive scale, negative scale and general scale of the PANSS showed greater improvement at T1 than at T0 (t=10.07, p<.001; t=12.39, p<.001; t=5.01, p<.001; t=9.58, p<.001). In addition, all symptoms, except for the negative symptoms (t=2.02, ns), were significantly improved at T2 in comparison to T0 (t=4.46, p<.001; t=6.51, p<.001; t=3.76, p<.01).

The changes in cognitive functioning over time were also analyzed (Table 3). Between T0 and T2, there were significant differences in the scores on the immediate list recall, immediate prose memory, and delayed name-face recall components of the K-MAS, but not in any of the other cognitive function assessments. Continuous evaluation of cognitive functioning throughout the treatment period showed that the subjects performed better on list learning, immediate list recall, delayed list recall, immediate prose memory, delayed prose memory, immediate name-face recall, delayed name-face recall, immediate visual recognition, verbal memory, visual memory and global memory components of the K-MAS and non-perseverative errors on the WCST at T1 than at T0. The patients showed greater improvement on memory assessments, such as the immediate list recall, delayed list recall, immediate prose memory, delayed prose memory and delayed name-face recall components of the K-MAS, at T2 in comparison to T0.

### Correlation between clinical symptoms and cognitive functions

The short-term memory component of the K-MAS and the total number of correct answers on the WCST were significantly correlated with positive symptoms (r=0.53, p<0.05; r=0.53, p<0.05). Total errors and nonperseverative errors on the WCST were significantly correlated with general symptoms (r=0.56, p<0.05; r=0.51, p<0.05). Nonperseverative errors on the WCST were also correlated with negative symptoms (r=0.51, p<0.05).

### Comparisons of cognitive function between treatment responders and non-responders

To investigate the predictive value of cognitive function in the progression of symptoms, the patient group was divided into treatment responders (n=20) and non-responders (n=14) at T2. The patients who were improved more than 20 percents in PANSS total score were classified as responders. The Kolmogorov-Smirnov test was applied to test for a normal distribution, and since it was demonstrated that distributions of two data were normalized, a parametric method was applicable.

Comparing cognitive functions between responders and non-responders at T0, responders showed significantly poorer performances in total correct answers, perseverative responses, perseverative errors, nonperseverative errors, conceptual level responses, and the number of category completed of WCST than non-responders (t=5.17, p<0.001; t=3.09, p<0.05; t=3.93, p<0.01; t=3.60, p<0.01; t=-2.67, p<0.05; t=-2.96, p<0.05). Comparing cognitive functions of responders with those of non-responders at T0-T2, significant differences appeared in immediate list recall, delayed list recall, immediate prose recall, and delayed name-face recall components of K-MAS (F=7.96, p<0.05; F=6.78, <0.05; F=4.71, p<0.05; F=7.39, p<0.05), and a significant interaction effect was revealed in total errors in the WCST (F=4.72, p<0.05) (Table 4).

### Discriminant analysis between responders and non-responders

Discriminant analysis was carried out to identify the cognitive domain most predictive of clinical improvement at T2. The changes in cognitive functioning were calculated following this formula: score at T0 minus score at T2. A total of 34 patients were analyzed. Of all the tests of cognitive functioning, the total number of errors on the WCST and the immediate list recall component of the K-MAS were most predictive of responder status, and the total number of errors on the WCST was the most important predictor (Table 5).

A single classification function was computed, and the result showed significant differences between responders and non-responders (Chi-square=13.004, df=2, p<0.01). A canonical correlation score of 0.833 demonstrated the high discriminant ability of this function. The immediate list recall component of the K-MAS was positively correlated with the discriminant function score. Therefore, it was supposed that the better list recall function, the better response. The total number of errors on the WCST was also negatively correlated with the discriminant function score, indicating that many errors in executive function might be associated with little improvement in clinical symptoms (Table 5). The classification function successfully predicted 82.4% of the patients' respondent status, correctly classifying 80% of responders and 85.7% of non-responders. Therefore, it was shown that the total number of errors on the WCST and the immediate list recall component of the K-MAS might discriminate between responders and non-responders.

## Discussion

Our results were consistent with the notion that cognitive dysfunction in patients with schizophrenia remains relatively static for the first few years after onset.^32^-^34^ This suggests that the cognitive dysfunction in patients with schizophrenia bears greater resemblance to a neurodevelopmental model than a neurodegenerative model and that the neuropsychological deficits in patients with schizophrenia are more stable than a neurodegenerative model would suggest. In addition, we observed consistent improvement in memory function over time. Understanding conversation and memorizing names and faces are essential to basic social adjustment, and our result demonstrated that medication had a positive effect on functional living in patients with schizophrenia.

Improvements in cognitive abilities, such as memory and executive function, were significantly correlated with the enhancement of clinical symptoms in patients with schizophrenia. These results confirmed that clinical symptoms and cognitive functions could be changed and enhanced by medication, and that the changes in symptoms and cognitive functions were not independent of each other, and acted reciprocally. In the present study, executive functioning showed significant correlations with positive, negative, and general symptoms. Stordal et al.^35^ showed that the general symptoms of schizophrenia were predictive of impairment in executive functioning. In this study, there was also a correlation between the total and nonperseverative errors on the WCST and general symptoms on the PANSS. However, other studies have suggested that executive function damage was highly relevant to negative symptoms of schizophrenia^36^-^38^ and aggravated social and occupational functions.^39^-^42^ It is also known that memory ability is significantly correlated with negative symptoms rather than positive symptoms.^43^,^44^ Most studies suggested that negative symptoms better explained cognitive impairments than positive symptoms. This study also found a lack of improvement in executive function and negative symptoms longitudinally. It did, however, identify significant correlations between negative symptoms and nonperseverative errors on the WCST for a short time. These results partially support those of previous studies.

The negative symptoms of schizophrenia include decreased ability to perform normal daily activities, alogia, blunted affect, anhedonia, and avolition. In the present study, there was no significant change in sustained attention, and the decrease in nonperseverative errors observed over a short period of 8 weeks was closely associated with test motivation. Clinical symptoms and cognitive functioning were improved dramatically at T1. This finding further demonstrated that the alleviation of psychotic symptoms influenced test performance. Nevertheless, one study that evaluated the cognitive functions of patients with schizophrenia for five years suggested that improvements in cognitive domains were associated with a decrease in positive symptoms but unrelated to negative symptoms. Thus, the relationship between clinical symptoms and cognitive functioning remains elusive.^15^

Changes in cognitive functioning emerged selectively in this study. That is, even if a patient's clinical symptoms improved, other cognitive functions, such as sustained attention and executive functions (except for nonperseverative errors on the WCST), did not show significant improvement. In fact, the results of the present study consistently demonstrated that a deficit in sustained attention was the main course of cognitive disturbance in patients with schizophrenia. Our results partially supported the idea that symptoms and cognitive functions are independent,^10^ and the cognitive functions of patients with schizophrenia are aggravated over time.^45^

The total number of errors on the WCST and immediate list recall component of the K-MAS were predictive of treatment response. Total errors on the WCST were a better predictor of treatment response than immediate list recall. Total errors included both perseverative errors and nonperseverative errors. The number of perseverative errors served as a reflection of difficulty in shifting cognitive sets according to various conditions. The number of perseverative errors was considered to be a significant cognitive variable for predicting patient relapse after their first episode.^19^

Similarly, perseverative errors also indicated that a perseverative response was a vulnerability indicator of schizophrenia.^46^ Social function has been related to social cognition,^47^ which may include emotional perception, social schema, insight, and coping strategies. Basic neurocognitive functions are required in order to understand the emotions of other people. If emotional perception ability is diminished, interpersonal relations prove be difficult.^48^ These data support that the effort to identify cognitive domains that can predict treatment response is of importance, as executive function is closely related to social adjustment.

The responders showed lower performance in terms of cognitive function, especially executive function, than the non-responders at T0. They also showed a longer duration of illness. It seems that as schizophrenia episodes repeat, the treatment response diminishes, and schizophrenia patients become recalcitrant to treatment and recovery.^49^,^50^ Therefore, the duration of illness influences the treatment response and cognitive performance. The cognitive functions of non-responders were mildly aggravated over time, regardless of treatment intervention. Unlike the responders, the non-responders failed to show improvement in clinical symptoms. Therefore, the improvement in clinical symptoms and clinical deficits that can be achieved by drug therapy is limited.

There were several methodological limitations of the present study. First, the subjects included in the present study were patients hospitalized in a specific psychiatric ward rather than patients from the local community. Therefore, our results may not be applicable to the entire population of persons suffering from schizophrenia. Second, we limited our comparison to patients with schizophrenia and normal controls.

To determine whether cognitive deficits implicated in schizophrenia are specific or represent other clinical variables, it is necessary to compare patients with schizophrenia to those with other mental disorders in a future study. A third limitation is that the subjects included in the present study were mostly paranoid schizophrenics with prominent positive symptoms.

## Figures and Tables

**TABLE 1:**
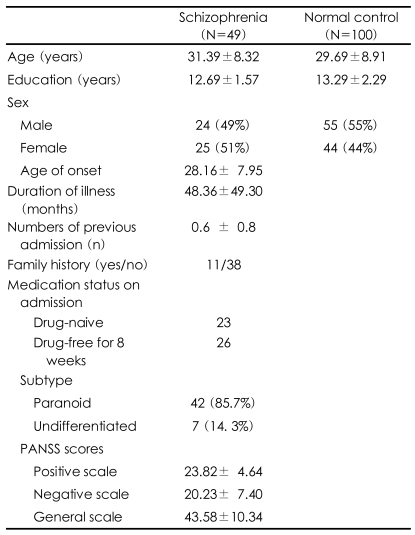
Demographic data for patients with schizophrenia and normal controls (mean±SD)

PANSS: Positive And Negative Syndrome Scale

**TABLE 2:**
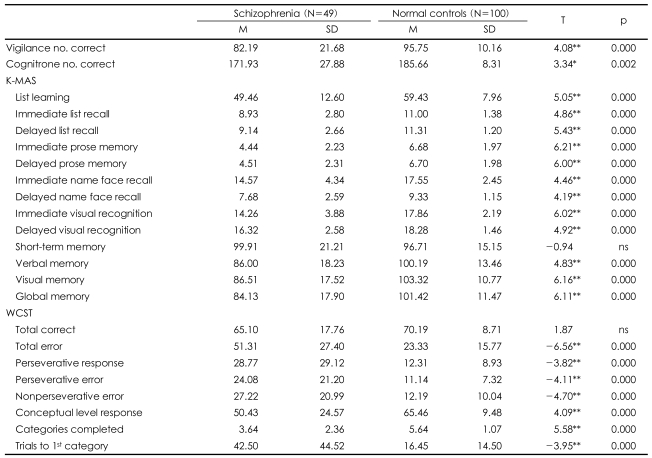
Comparisons of cognitive functions between schizophrenia patients and normal controls

^*^p<0.01, ^**^p<0.001. K-MAS: Korean version of the Memory Assessment Scales, WCST: Wisconsin Card Sorting Test

**TABLE 3:**
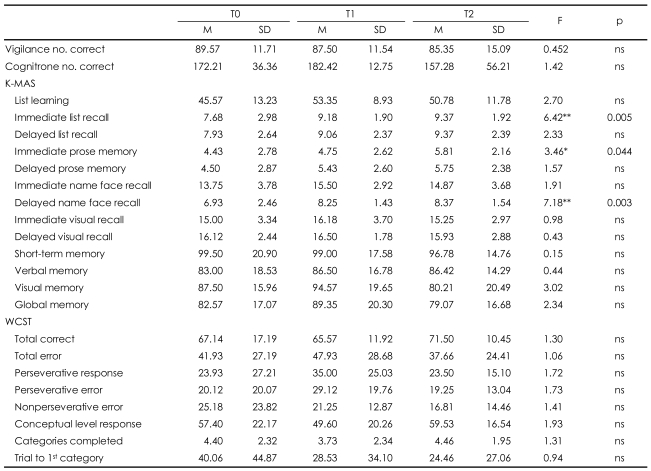
Mean and SD of cognitive scores in schizophrenia patients (N=34)

^*^p<0.05, ^**^p<0.01. T0: before antipsychotic treatment, T1: eight weeks after treatment, T2: one year after treatment, K-MAS: Korean version of the Memory Assessment Scales, WCST: Wisconsin Card Sorting Test

**TABLE 4:**
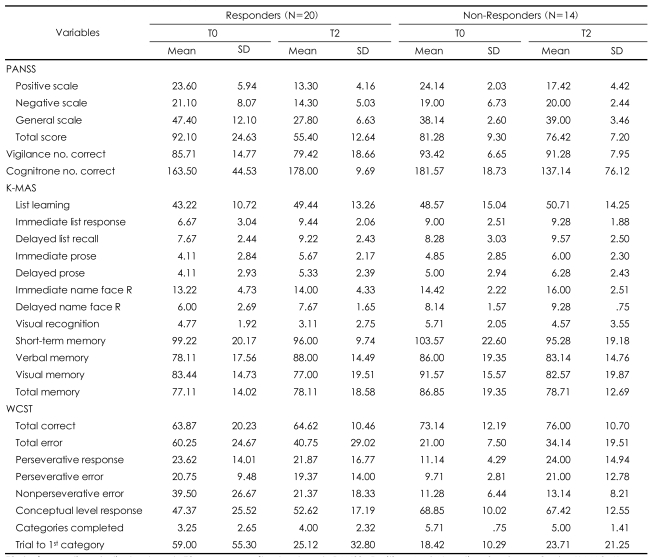
Mean and SD of clinical and cognitive scores between responders and non-responders

T0: before antipsychotic treatment, T2: one year after treatment, PANSS: Positive And Negative Syndrome Scale, K-MAS: Korean version of the Memory Assessment Scales, WCST: Wisconsin Card Sorting Test

**TABLE 5:**
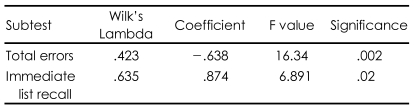
Discriminant analysis between responders and non-responders
